# Classical complement cascade initiating C1q protein within neurons in the aged rhesus macaque dorsolateral prefrontal cortex

**DOI:** 10.1186/s12974-019-1683-1

**Published:** 2020-01-06

**Authors:** Dibyadeep Datta, Shannon N. Leslie, Yury M. Morozov, Alvaro Duque, Pasko Rakic, Christopher H. van Dyck, Angus C. Nairn, Amy F. T. Arnsten

**Affiliations:** 1 Department of Neuroscience, Yale University School of Medicine, 333 Cedar St., New Haven, CT 06511 USA; 2 Department of Psychiatry, Yale University School of Medicine, New Haven, USA; 3 Interdepartmental Neuroscience Program, Yale University School of Medicine, New Haven, USA

**Keywords:** Prefrontal cortex, Complement C1q, Aging, Pyramidal cell, Microglia, cAMP

## Abstract

**Background:**

Cognitive impairment in schizophrenia, aging, and Alzheimer’s disease is associated with spine and synapse loss from the dorsolateral prefrontal cortex (dlPFC) layer III. Complement cascade signaling is critical in driving spine loss and disease pathogenesis. Complement signaling is initiated by C1q, which tags synapses for elimination. C1q is thought to be expressed predominately by microglia, but its expression in primate dlPFC has never been examined. The current study assayed C1q levels in aging primate dlPFC and rat medial PFC (mPFC) and used immunoelectron microscopy (immunoEM), immunoblotting, and co-immunoprecipitation (co-IP) to reveal the precise anatomical distribution and interactions of C1q.

**Methods:**

Age-related changes in C1q levels in rhesus macaque dlPFC and rat mPFC were examined using immunoblotting. High-spatial resolution immunoEM was used to interrogate the subcellular localization of C1q in aged macaque layer III dlPFC and aged rat layer III mPFC. co-IP techniques quantified protein-protein interactions for C1q and proteins associated with excitatory and inhibitory synapses in macaque dlPFC.

**Results:**

C1q levels were markedly increased in the aged macaque dlPFC. Ultrastructural localization found the expected C1q localization in glia, including those ensheathing synapses, but also revealed extensive localization within neurons. C1q was found near synapses, within terminals and in spines, but was also observed in dendrites, often near abnormal mitochondria. Similar analyses in aging rat mPFC corroborated the findings in rhesus macaques. C1q protein increasingly associated with PSD95 with age in macaque, consistent with its synaptic localization as evidenced by EM.

**Conclusions:**

These findings reveal novel, intra-neuronal distribution patterns for C1q in the aging primate cortex, including evidence of C1q in dendrites. They suggest that age-related changes in the dlPFC may increase C1q expression and synaptic tagging for glial phagocytosis, a possible mechanism for age-related degeneration.

## Background

The newly evolved dorsolateral prefrontal cortex (dlPFC) subserves our highest-order cognitive functions, generating the mental representations that are the foundation for abstract thought, higher reasoning, goal-directed behavior, and executive functions [1]. The dlPFC is a target of pathology in schizophrenia, aging, and Alzheimer’s disease (AD), including spine loss in the layer III recurrent excitatory circuits that underlie the working memory in human and non-human primates [1–6]. These layer III circuits are particularly vulnerable to atrophy due to their unique molecular regulation [7], including dysregulated feedforward calcium-cAMP signaling in aged monkeys associated with reduced neuronal firing [8], tau phosphorylation [9], and mitochondrial abnormalities [10, 11].

Age-related changes may initiate inflammatory complement cascades that drive phagocytosis of synaptic connections on spines and dendritic arbors. The classical complement cascade signaling pathway has a crucial role in defense from pathogens (e.g., bacteria) and removal of cellular debris [12, 13]. However, a plethora of discoveries purports a role of complement cascade signaling in synapse pruning during postnatal development, disease-associated synapse elimination, and cognitive impairments with advancing age [12, 14, 15]. The classic complement cascade is initiated by C1q, which induces a complex sequence of downstream events (C2–4 and their receptors). For example, mice deficient in C1q or C3 exhibit sustained defects in the pruning of retinogeniculate synapses in the developing visual system [15]. C1q and C3 concomitantly “tag” vulnerable synaptic elements, which are then engulfed by microglia using the cognate receptor CR3. There is also a dramatic upregulation of synapse-associated C1q during aging in the rodent cortex, which plays a role in age-related memory dysfunction [16]. Aberrant re-activation of complement cascade signaling pathways also has been implicated in various neuropsychiatric and neurodegenerative disorders [13, 17]. In mouse models of AD, C1q is necessary for soluble β-amyloid oligomers to induce synapse elimination before overt plaque formation [18]. Similarly, robust aggregation of C1q has been observed near the postsynaptic density (PSD) of Tau-P301S mice and in postmortem AD brain, alterations that are associated with the microglial engulfment of synaptic elements [19]. C3 and C3a receptor (C3aR1) are also positively correlated with cognitive decline and Braak tau staging in human AD brains [20]. Conversely, attenuation of complement cascade signaling pathways using genetic and/or antibody-mediated suppression of C1q leads to the rescue of synaptic deficits, neuroinflammation, and pathological phenotypes [18–20]. Finally, GWAS studies have found that alterations in complement C4a are associated with the greatest risk for schizophrenia [21]. Thus, there are multiple studies indicating that complement signaling drives microglial engulfment of synapses in various conditions and disorders.

However, we have limited understanding of how complement cascade proteins impinge on cortical circuits in a cell type-specific fashion and no knowledge of their precise subcellular location. Studies of the brains from mouse AD models have proffered that C1q is predominately expressed in the microglia, with additional, but very limited, expression in interneurons [22]. The near-exclusive expression of C1q in the microglia was also seen in the aged mouse brain [22], suggesting that neurons play a minor role in initiating complement signaling in the aged brain. Given the importance of prefrontal cortex (PFC) circuits to human cognition and cognitive disorders, the current study examined the ultrastructural localization of complement cascade signaling initiating protein C1q in the aged rhesus monkey dlPFC, focusing on the layer III microcircuits with known spine loss. We also used biochemical methods to quantify and assess protein-protein interactions for C1q in young and aging rhesus macaque dlPFC. Parallel studies were conducted in the aging rat medial PFC (mPFC), the PFC subregion needed for working memory in rodents [23]. As with monkeys, rats develop working memory deficits [24] and spine loss from layer III mPFC [25] with advanced age, and thus may exhibit similar changes in complement signaling. This study found the expected rise in C1q levels with age in rats and monkeys, with pronounced localization in the glia, but also found an unexpected localization of C1q within pyramidal cells, and a significant interaction between C1q and PSD95 that increased with age, consistent with its intra-neuronal expression in the aging PFC.

## Methods

Rhesus monkeys (*Macaca mulatta*) and Sprague-Dawley rats were maintained and euthanized in accordance with the guidelines of Yale University Institutional Animal Care and Use Committee and National Institutes of Health “Guidelines for the Care and Use of Experimental Animals”.

### Electron microscopy

#### Animals and tissue processing

**Fig. 1:**
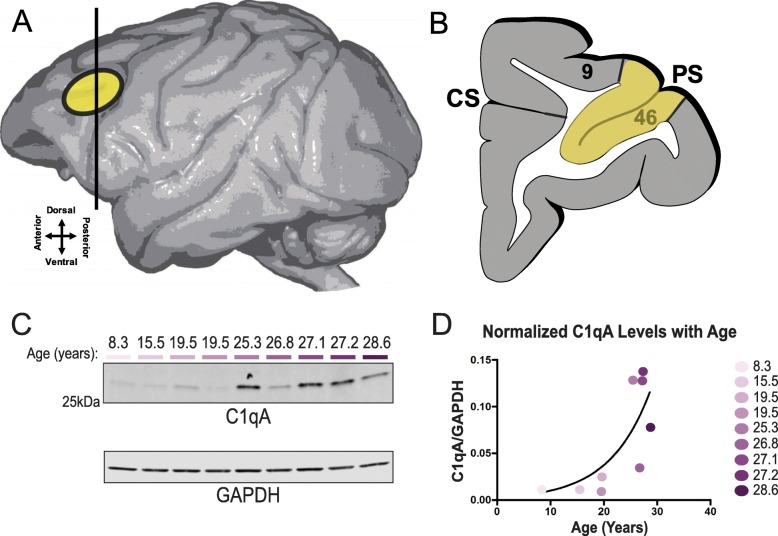
Graphical illustration of the region sampled for ultrastructural and biochemical studies and age-related increases in C1q protein expression. **a** The region sampled for immunocytochemical, ultrastructure, and biochemistry studies. Schematic depicting the rhesus macaque brain and region (yellow oval) showing the location of the dorsolateral prefrontal cortex. The entire region was excised for biochemistry analyses to evaluate C1q protein expression. Approximately 60-μm-thick sections were cut for subsequent pre-embedding immunocytochemical labeling of C1q. **b** Cartoon illustrating the location of Walker’s area 46 and the principal sulcus (PS), which constitute the dlPFC and used for analyses in this study (highlighted in yellow). **c**, **d** Biochemical characterization of C1qA in macaque cortical tissue. Triton soluble macaque dlPFC tissue was immunoblotted for C1qA and GAPDH. Quantification of C1qA increase in macaque dlPFC with age was fit with an exponential growth curve (*R*^2^ = 0.5473). Each animal is color-coded by age

Three aged (26–28 years) rhesus monkeys and two aged (27 and 29 months) Sprague-Dawley rats were used for immunoelectron microscopy (immunoEM). As described previously [26], the animals were deeply anesthetized prior to transcardial perfusion of the artificial cerebrospinal fluid, followed by 4% paraformaldehyde/0.05% glutaraldehyde in 100 mM phosphate-buffered saline. Following perfusion, a craniotomy was performed, and the entire brain was removed and dissected, including a frontal block containing the primary region of interest surrounding the principal sulcus. The brains were sectioned coronally at 60 μm on a vibratome (Leica) across the entire rostrocaudal extent of the dorsolateral prefrontal cortex (dlPFC; Walker’s area 46) for macaques and medial prefrontal cortex (mPFC) for rats. The sections were cryoprotected through increasing concentrations of sucrose solution (10%, 20%, and 30% each for 2 h, then 30% overnight), cooled rapidly using liquid nitrogen, and stored at − 80 °C. Sections of macaque dlPFC and rat mPFC were processed for C1q immunocytochemistry (Fig. 1a, b). In order to enable penetration of immunoreagents, all sections went through three freeze-thaw cycles in liquid nitrogen. Non-specific reactivity was suppressed with 10% normal goat serum (NGS) and 2% bovine serum albumin (BSA), and antibody penetration was enhanced with 0.3% Triton X-100 in 50 mM Tris-buffered saline (TBS).

#### Histology and immunoreagents

An extensively characterized mouse monoclonal C1q (JL-1) antibody reactive against the collagen-like region (CLR) obtained commercially (ab71940; Abcam, Cambridge, MA, USA) was used for immunoEM studies at 1:200 dilution. The antibody was generated by immunization of C1q^−/−^ C57BL/6 mice with purified mouse C1q. The antibody has been previously shown to recognize C1q with a high degree of specificity in a myriad of different tissues and cell types by immunoblotting, immunohistochemistry, and immunofluorescence procedures [27–30]. The C1q antibody detects a band migrating at ~ 26 kDa in immunoblots. Normal sera and IgG-free BSA were purchased from Jackson Immunoresearch (West Grove, PA, USA). All chemicals and supplies for electron microscopy were purchased from Sigma Aldrich (St. Louis, MO, USA) and Electron Microscopy Sciences (Hatfield, PA, USA), respectively.

#### Single pre-embedding peroxidase immunocytochemistry

3,3-Diaminobenzidine tetrahydrochloride (DAB) labeling was used in order to enhance the detection sensitivity and to have access within the PSD using the pre-embedding technique, which preserves the plasma and intra-neuronal membranes. As described previously [31], the sections were incubated for 72 h at 4 °C with C1q antibody in TBS and transferred for 2 h at room temperature to species-specific biotinylated Fab′ or F (ab′)_2_ fragments in TBS. In order to reveal immunoperoxidase labeling, the sections were incubated with the avidin-biotin peroxidase complex (ABC) (1:300; Vector Laboratories, Burlingame, CA, USA) and then visualized in 0.025% Ni-intensified DAB (Sigma Aldrich, St. Louis, MO, USA) as a chromogen in 100 mM PB with the addition of 0.005% hydrogen peroxide for 10 min. After the DAB reaction, the sections were exposed to osmification, dehydration, and standard resin embedding following the standard immunoEM procedures. Omission of C1q primary antibody or substitution with non-immune serum resulted in the complete lack of immunoperoxidase labeling.

#### Electron microscopy and data analysis

All sections were processed as previously described [31]. Briefly, blocks containing macaque dlPFC layer III and rat mPFC layer II/III were sampled and mounted onto resin blocks. The specimens were cut into 50-nm sections using an ultramicrotome (Leica, Norcross, GA, USA) and analyzed under a JEM1010 (Jeol, Tokyo, Japan) transmission electron microscope at 80 kV. Several plastic blocks of each brain were examined using the 4th to 12th surface-most sections of each block (i.e., 200–600 nm), in order to sample the superficial component of the sections, avoiding penetration artifacts. Structures were digitally captured at × 25,000–100,000 magnification (Gatan, Pleasanton, CA, USA), and individual panels were adjusted for brightness and contrast using Adobe Photoshop and Illustrator CC.2017.01 image editing software (Adobe Systems Inc., San Jose, CA, USA). Approximately, 1000 micrographs of the selected areas of neuropil with immunopositive profiles were used for analyses. For profile identification, we adopted the criteria summarized by Peters and colleagues [32, 33]. Dendritic spines in the PFC are typically long and thin, devoid of mitochondria with the presence of a well-developed postsynaptic density (PSD) at asymmetric synapses. Dendritic shafts were typically round in perpendicular planes or irregularly shaped when assessed in horizontal planes, usually containing mitochondria and numerous tubular and pleomorphic cellular organelles. Depending on the proximity to axon terminals, various dendritic shafts received synaptic inputs. Axon terminals contained accumulations of synaptic vesicles and the axoplasm of these terminals usually contained neurofilaments and mitochondria. The synaptic innervations made by these axon terminals were either asymmetric, containing spherical vesicles, or symmetric, containing pleomorphic vesicles, with typical differences in PSD. Unmyelinated axons were small, round processes with a predominantly even and regular shape, traversing the neuropil in a straight orientation, often forming bundles in perpendicular planes. Astroglial processes were typically of irregular morphology, forming contours that filled the empty space around neuronal elements.

### Biochemistry

#### Animals and tissue processing

Monkeys used for biochemical and co-IP experiments ranged in age from 8.3 to 28.6 years (*N* = 9 animals). Due to the rarity of acquiring fresh tissue from aging macaques, the subjects had varied medical and health histories, similar to human postmortem studies. Following removal of the dura, great care was taken to remove dlPFC tissue using a scalpel, minimizing the postmortem interval. Immediately following dissection, samples were placed into liquid nitrogen and stored at − 80 °C long term. Samples analyzed in this study were collected between July 2010 and March 2019.

Rats for biochemical experiments were handled at least four times prior to euthanasia in order to minimize stress. Young rats were sacrificed at roughly 3.5 months of age (*N* = 10). Aged rats were closely monitored once they reached 20 months of age for any signs of health deterioration. At the first sign of altered behavior, animals were euthanized. Based on these criteria, aged rats ranged from 24.5 to 30 months of age, averaging 27.8 months (*N* = 10 animals). In order to further investigate the changes at extreme ages, aged animals were divided into two cohorts: those younger than 28 months of age (*N* = 5) and those older than 28 months of age (*N* = 5). This separation was based on the large age range for aged animals compared to no variation in age of young animals as well as the observation of an exponential rise in C1q with advancing ages in macaques, indicating that changes in rats may only be visible in very old animals. For euthanasia, rats were briefly anesthetized with isoflurane and then rapidly decapitated with a guillotine. The brain was quickly removed, and the dissection began as soon as possible to minimize postmortem interval. The front cortex block of tissue was separated with a razor blade, and the two hemispheres were split to speed the freezing process. The tissue was immediately frozen on dry ice and stored at − 80 °C until all of the rat tissue was collected. Samples used in this study were collected between December 2018 and June 2019.

#### Co-immunoprecipitation

Tissue lysate preparation was based on previously described methods [19]. Frozen tissue was homogenized in immunoprecipitation buffer (5 mM HEPES, 1 mM MgCl_2_, 0.5 mM CaCl_2_, phosSTOP phosphatase inhibitor, and cOmplete mini protease inhibitor) utilizing a rotor homogenizer. Lysate was centrifuged at 1000*g* for 5 min at 4 °C to pre-clear the nuclei. The supernatant was then centrifuged at 10,000*g* for 10 min at 4 °C. The pellet was resuspended in immunoprecipitation buffer with 0.5% Triton X-100 and cleared at 10,000*g* for 10 min. The triton soluble lysate was incubated overnight with 10 μg of the immunoprecipitation antibody coupled to dynabeads (PSD95 or Gephyrin as specified). A control IgG immunoprecipitation was also run with no effective pulldown of the targets of interest. The beads were washed three times for 5 min in immunoprecipitation buffer with 0.5% Triton X-100 and eluted in 1% SDS with protein loading buffer by boiling.

#### Immunoblotting

Triton soluble samples were rotor homogenized in 1% Triton X-100 lysis buffer (200 mM NaCl, 10 mM HEPES, 10 mM EGTA, 10 mM EDTA, phosSTOP phosphatase inhibitor, and cOmplete mini protease inhibitor) and pre-cleared by 5 min > 15,000*g* centrifugation at 4 °C. All protein samples were boiled in SDS loading buffer with DTT. Samples were run on 4–20% Tris-glycine gels and transferred onto 0.2-μm nitrocellulose membranes. The membranes were blocked for 1 h with 5% milk. Primary antibodies were prepared in LI-COR blocking buffer (PSD95 CST #3450 1:1000; Gephyrin Chemicon AB5725 1:1000; GAPDH Millipore CB1001-500 1:10,000; C1qA Abcam ab189922 1:500) and incubated overnight at 4 °C. Fluorescent secondary antibodies of the appropriate species were prepared in LI-COR blocking buffer and incubated for 1 h at room temperature. All washes were done in PBS with 0.1% Tween. Blots were analyzed utilizing a LI-COR Odyssey scanner. Quantification of bands was done in ImageStudio Lite with background subtraction calculated by the average intensity immediately above and below the band of interest.

#### Statistical analysis

All protein levels were normalized to loading or immunoprecipitation control prior to the analysis as described in figure legends. Macaque C1qA values were fit with a non-linear exponential growth model. Rat frontal cortex block samples were analyzed in three groups (young, aged 1 < 28 months, and aged 2 > 28 months). Values were compared using Dunnett’s multiple comparisons test.

## Results

The levels and locations of C1q were examined in the aging macaque dlPFC and rat mPFC.

### C1q Expression in aging rhesus macaque dlPFC

#### Increased C1qA expression with advancing age

Given previous reports of increased C1q expression in aging rodent brain [16, 34], we examined the levels of expression of C1q in the rhesus monkey dlPFC across the adult age span using immunoblot analyses. These experiments revealed a striking increase in C1q levels with age, which was well modeled by an exponential growth curve (*R*^2^ = 0.5473) (Fig. 1c, d). The older age range (greater than 20 years), which had the highest levels of C1q expression, corresponds to the age range when spine loss is found in macaque dlPFC layer III [3, 33, 35].

#### ImmunoEM localization in aged macaque layer III dlPFC

**Fig. 2:**
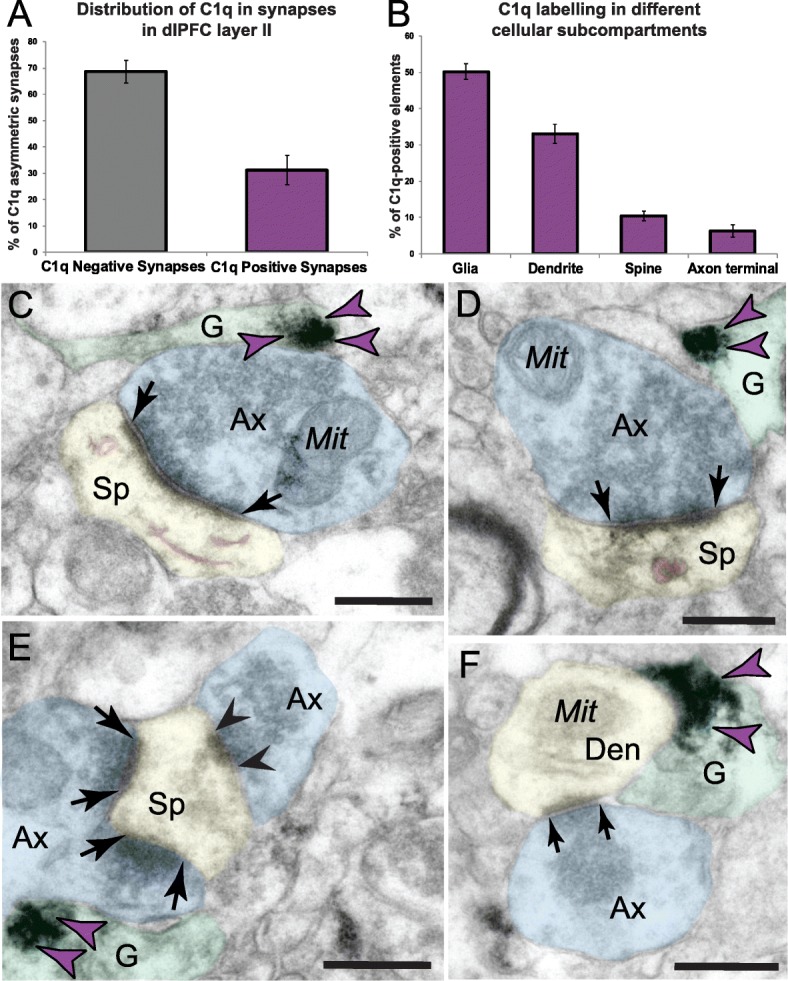
Quantitative distribution of C1q immunolabeling in aged macaque dlPFC layer III and labeling in glial profiles. The prevalence of C1q labeling in asymmetric synapses in dlPFC layer III neuropil, expressed as a percentage (**a**). Ultrastructural localization of C1q within different cellular elements in aged monkey dlPFC layer III. The percentage of immunoperoxidase labeling (mean ± SEM) for C1q across glia, dendritic shafts, dendritic spines, and axon terminals in aged macaque dlPFC layer III, *N* = 3 aged rhesus macaques (**b**). Immunolabeling for C1q is present along the plasma membrane and within the intracellular space of glial processes. **c**–**e** C1q labeling is observed in glial leaflets ensheathing the axospinous glutamatergic-like asymmetric synapses. **c** C1q labeling is observed in glial leaflets ensheathing a synaptic triad or dually innervated spine, receiving a glutamatergic-like asymmetric synapse with the axon terminal containing spherical neurotransmitter vesicles and a symmetric synapse (black arrowhead) with the axon terminal containing pleomorphic neurotransmitter vesicles (likely GABA or dopamine). The smooth endoplasmic reticulum (SER) spine apparatus is pseudocolored in pink. **f** C1q labeling is also seen in glial leaflets ensheathing axodendritic glutamatergic-like synapses. Synapses are between black arrows. Color-coded arrowheads (purple) point to C1q immunoreactivity. The profiles are pseudocolored for clarity. Ax, axon; Sp, spine; Den, dendrite; Mit, mitochondria; G, glia. Scale bars, 200 nm

ImmunoEM was used to determine the ultrastructural localization of C1q in the aged layer III dlPFC circuits with established, age-related spine loss from pyramidal cells [3, 33]. Across all aged animals, out of 278 synapses analyzed, C1q was deposited in 87 sites (31.29%), predominantly in postsynaptic locations, but also in presynaptic compartments (Fig. 2a). We conducted an extensive analysis to identify C1q distribution in different cellular elements. We analyzed 460 C1q immunopositive profiles across different cell types in dlPFC layer III. The distribution by cellular elements is as follows: glia (231 sites; 50.22%), dendrite (152 sites; 33.04%), dendritic spines (48 sites; 10.43%), and axon terminals (29 sites; 6.30%) (Fig. 2b).

##### Localization of C1q in glial processes

**Fig. 3:**
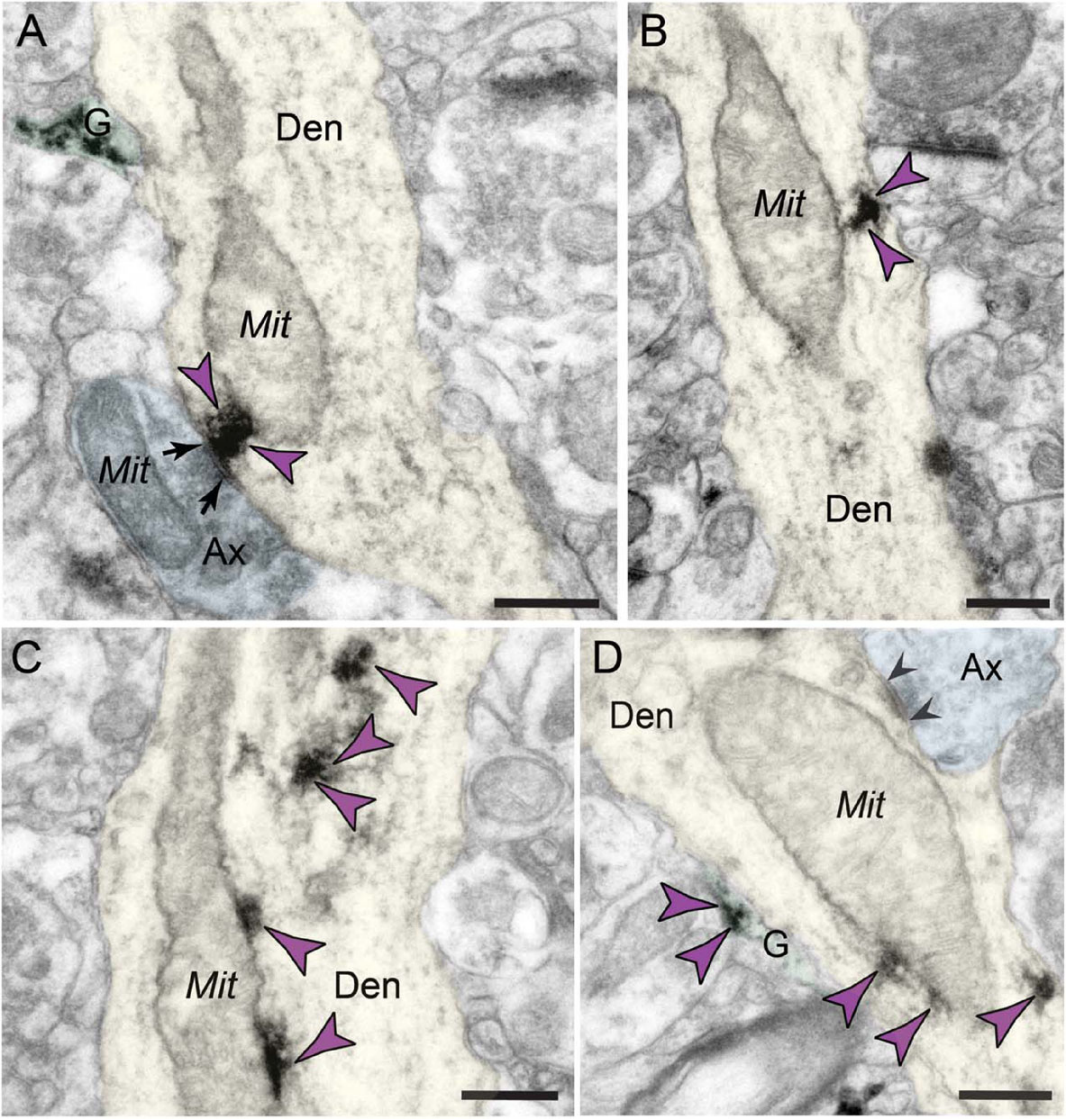
Extensive labeling of C1q within dendritic shafts in postsynaptic compartments in aged monkey dlPFC layer III. **a**–**d** C1q immunolabeling is found accumulating in the dendritic shafts, and the label is often associated with abnormal mitochondria-on-a-string (MOAS) profiles in aged monkey dlPFC layer III. **a** The immunolabeling shows association with synapses and postsynaptic density and associated with MOAS-like profile. **b** The immunolabeling is associated with the inner aspect of the shaft plasmalemma, and the label appears subjacent to the plasma membrane. **c**, **d** C1q labeling is visualized in association with microtubules in the intracellular space, in close proximity to MOAS-like profiles. Synapses are between arrows and arrowheads (symmetric synapses). Color-coded arrowheads (purple) point to C1q immunoreactivity. Profiles are pseudocolored for clarity. Ax, axon; Den, dendrite; Mit, mitochondria; G, glia. Scale bars, 200 nm

C1q protein localization was prominent in aged dlPFC layer III glial processes (Fig. 2). Glial expression of C1q was present on the plasma membrane and within the intracellular space. Labeling was often visualized in glial leaflets ensheathing axospinous inputs, receiving glutamatergic-like asymmetric synapses (Fig. 2c–e). C1q was not uniformly distributed along the membranes but showed greater specificity for labeling next to the synapse interface in the perisynaptic subcompartments. We also observed C1q-labeled glial processes near axodendritic glutamatergic-like synapses, in close proximity to the dendritic mitochondria (Figs. 2f and 3a, d). These findings corroborate previous immunohistochemistry, RNA sequencing, and genetic ablation studies in rodents that have identified glial cells as the predominant cell type for C1q expression and localization [16, 22, 36–39].

##### Postsynaptic localization in dendritic shafts

We found C1q labeling in postsynaptic neuronal compartments in monkey dlPFC layer III (Figs. 2a, b and 3). A prominent postsynaptic localization of C1q was in dendritic shafts. C1q immunoreactivity was observed in close proximity to dysmorphic mitochondria (Fig. 3a). We have previously described the presence of abnormal mitochondria-on-a-string (MOAS) profiles in aged monkey dlPFC layer III, typified by enlarged profiles, interconnected by highly constricted segments, indicative of impairments in mitochondrial fission/fusion [11]. C1q was observed next to the mitochondria with the MOAS phenotype (Fig. 3a–d), in association with the outer mitochondria membrane (OMM; Fig. 3a–d). C1q was also observed subjacent to the plasma membrane near the abnormal mitochondria (Fig. 3b), and possibly on the nearby smooth endoplasmic reticulum (SER) and/or microtubules where it may traffic to other subcompartments (Fig. 3c, d).

##### Postsynaptic localization in dendritic spines

**Fig. 4:**
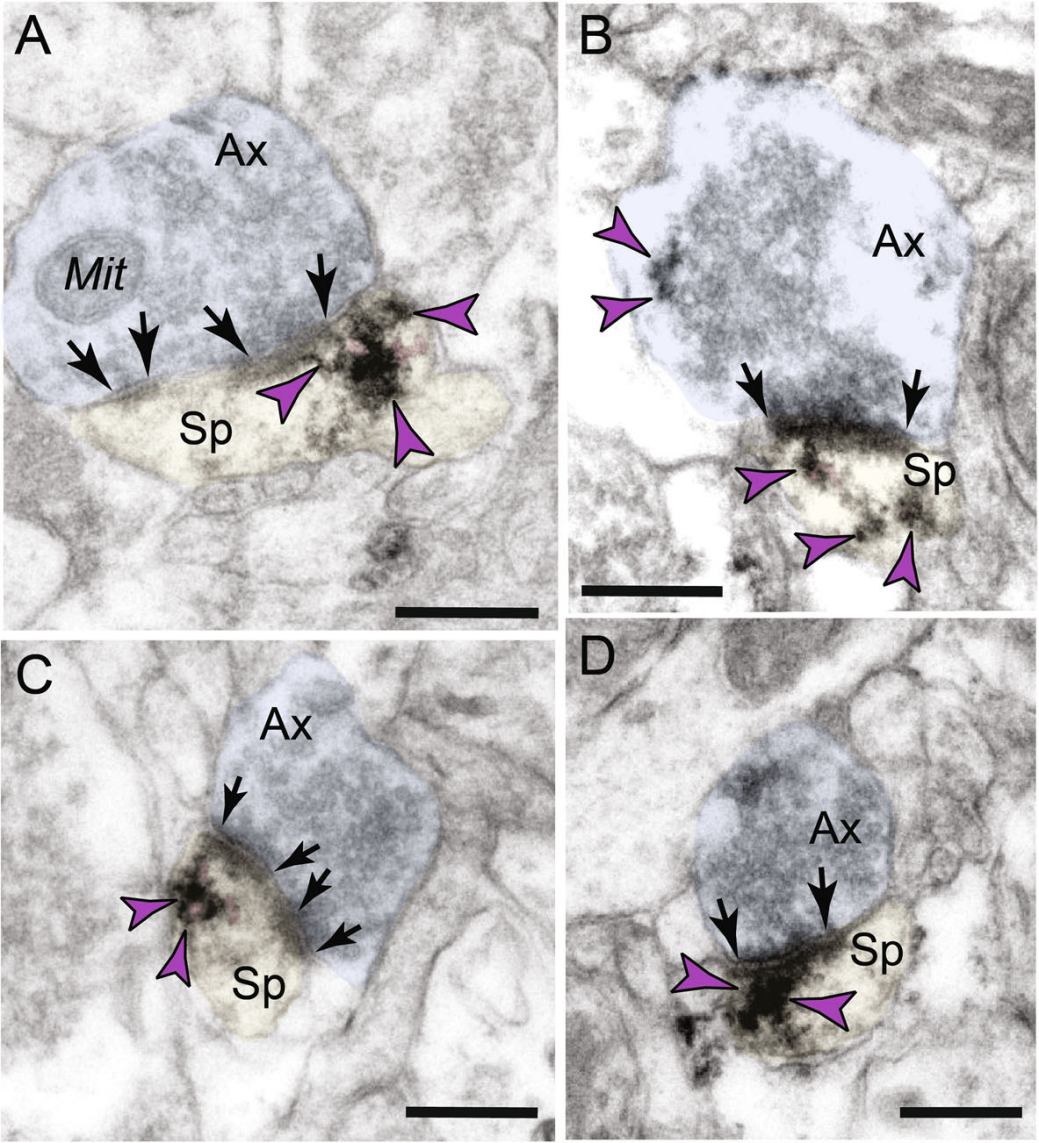
Intra-neuronal labeling of C1q within dendritic spines in aged monkey dlPFC layer III. **a**–**d** Postsynaptic C1q in dendritic spines in aged monkey dlPFC is prominently associated with the smooth endoplasmic reticulum (SER) spine apparatus (pink pseudocolored) and is observed in perisynaptic or extrasynaptic locations near the PSD. **d** C1q protein is also visualized within the glutamatergic-like PSD per se. Synapses are between arrows. Color-coded arrowheads (purple) point to C1q immunoreactivity. Profiles are pseudocolored for clarity. Ax, axon; Sp, spine; Mit, mitochondria. Scale bars, 200 nm

Ultrastructural localization revealed a robust accumulation of C1q in mature, thin-type dendritic spines within neurons in aged monkey dlPFC layer III (Figs. 2a, b and 4). Postsynaptic C1q in dendritic spines was associated with, or captured next to, the SER spine apparatus, where it may be influenced by dysregulated signaling (Fig. 4a–c). C1q labeling was also observed in perisynaptic or extrasynaptic locations near the PSD (Fig. 4a, c) or within the glutamatergic-like PSD per se (Fig. 4d). In particular, C1q exhibited non-uniform expression along the length of the synaptic active zone. C1q labeling in the spines was also associated with the plasma membrane (e.g., Fig. 4a, c), potentially signaling to the microglia to eliminate vulnerable neuronal elements by phagocytosis.

##### Presynaptic localization in axon terminals

**Fig. 5:**
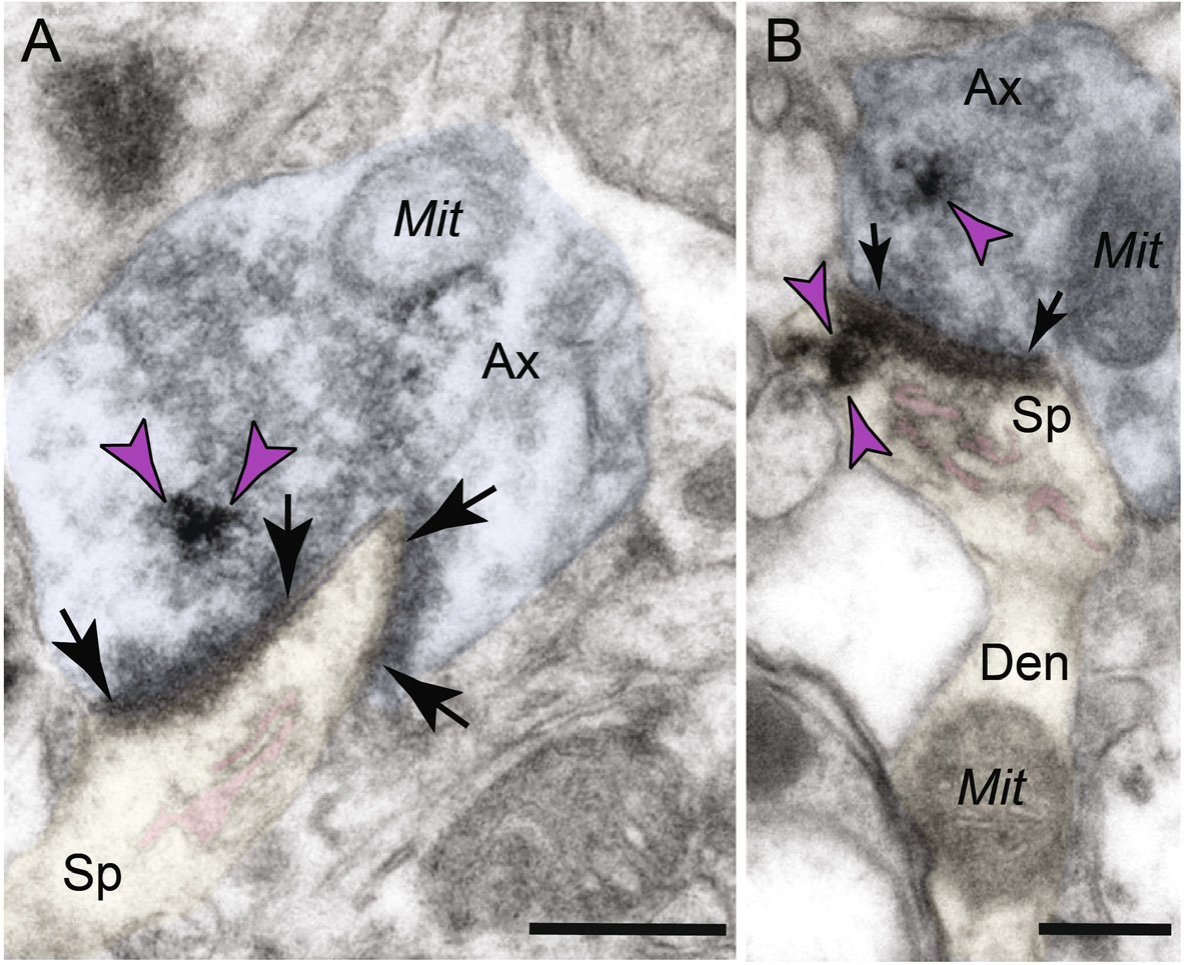
Axon terminal C1q labeling is observed in association with synaptic vesicles. **a**, **b** Presynaptic C1q is visualized in association with vesicles within glutamatergic-like axon boutons and rarely on the plasma membrane. **a** C1q is localized in axon terminals in association with spherical vesicles. The perforated asymmetric synapse can be seen clearly, along with a distinct spine apparatus in the postsynaptic compartment. **b** C1q labeling is observed in both pre- and postsynaptic compartments of the same synaptic profile. The dendritic spine head of this profile can be seen emanating from a dendritic shaft containing mitochondria. Synapses are between arrows. Color-coded arrowheads (purple) point to C1q immunoreactivity. Profiles are pseudocolored for clarity. Ax, axon; Sp, spine; Mit, mitochondria. Scale bars, 200 nm

In addition to its postsynaptic localization, C1q was sparsely localized in glutamatergic-like axon terminals (Figs. 2a, b and 5). Presynaptic C1q was visualized in association with synaptic vesicles, but not on the plasma membrane (Fig. 5a, b). In contrast to the spines, the labeling pattern in presynaptic axon terminals was not observed perisynaptically or extrasynaptically, nor near the axonal plasma membrane. Intriguingly, C1q immunoreactivity could be observed simultaneously in both presynaptic and postsynaptic compartments of the same synaptic profile, receiving a glutamatergic-like asymmetric synapse (Fig. 5b).

#### Immunoprecipitation of C1q with synaptic proteins

**Fig. 6:**
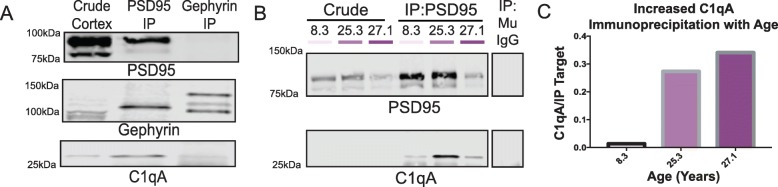
Increased co-immunoprecipitation of C1q with PSD95 and gephyrin. **a** PSD95 (5 μg antibody) or gephyrin (10 μg antibody) was immunoprecipitated from 300 μg cortical tissue of the oldest macaque. The immunoprecipitated material was blotted for PSD95, gephyrin, and C1qA. **b** PSD95 was immunoprecipitated from the dlPFC of three animals along with a mouse IgG control (400 μg starting tissue in 8.3 and 25.3-year-old animals and 250 μg starting tissue in 27.1-year-old animal, mu IgG control was run on a pool of all three animal lysates ~ 250 μg). Immunoprecipitated elution was blotted for PSD95 and C1qA. **c** Quantification of C1qA levels co-immunoprecipitated with PSD95 represented as a ratio of C1qA over PSD95. The graph is color-coded by animal age as denoted in Fig. 1d

Given the close association of C1q with the asymmetric synapse on the spines, we tested for C1q immunoprecipitation with the excitatory synapse marker PSD95 and the inhibitory synapse marker, gephyrin, using newly lysed dlPFC tissue from the oldest macaque. Previous reports from tauopathy mouse models revealed selectivity for C1q interaction with PSD95 and not gephyrin [19]. We found that C1q preferentially immunoprecipitated with PSD95 from macaque dlPFC (Fig. 6a), consistent with the immunoEM showing C1q labeling in glutamate-like synapses on spines (Fig. 2a, b).

A second experiment examined age-related changes in the C1q interactions with PSD95 from three animals spanning the age range of our cohort. This pulldown showed an age-dependent increase in the ratio of C1qA that co-immunoprecipitated with PSD95 (Fig. 6b, c). Together, these results suggest that C1q levels increase with age in rhesus macaques at excitatory synapses known to be susceptible to degeneration, consistent with the pattern of immunoEM labeling and the vulnerability of dlPFC spines to atrophy with advancing age.

### C1Q expression in aging rat mPFC

Given the striking changes in C1q seen in aging monkeys, we examined the changes in a simpler model system, the rat, in order to understand whether these observations were conserved across species.

#### Increased C1qA expression in very advanced age in rats

**Fig. 7:**
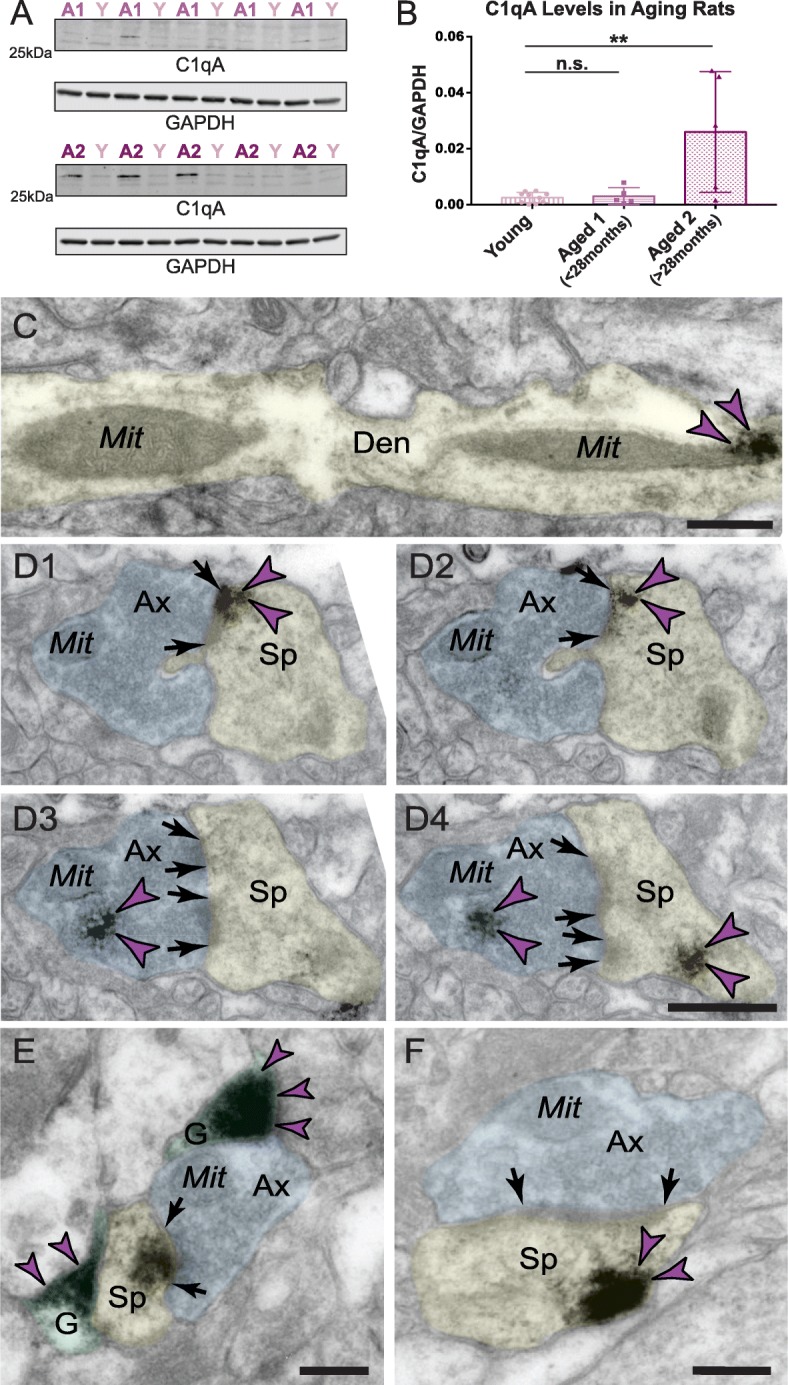
Age-related alterations in C1q protein in rat medial PFC. Biochemical characterization of C1qA in the rat frontal cortex. **a** Triton-soluble fractions from rat frontal cortex blocks were immunoblotted for C1qA and GAPDH. Lane labels are color-coded by age group; Y = young, ~ 3.5 months (*n* = 10); A1 = aged < 28 months (*n* = 5); A2 = aged > 28 months (*n* = 5). **b** Quantification of C1qA increase in the rat frontal cortex in the most advanced age group. Dunnett’s multiple comparison test to the young age group (adjusted *p* values: Y vs. A1 *p* = 0.995, Y vs. A2 **p = 0.002). **c** Significant labeling of C1q within dendritic shafts in postsynaptic compartments in aged rat mPFC layer II/III in close proximity to MOAS profiles. **d1**–**d4** Intra-neuronal pre- and postsynaptic labeling of C1q protein in axospinous asymmetric synapse in aged rat mPFC layer II/III. The C1q immunopositive axospinous synapse is visualized in serial sections in rat mPFC sections. Postsynaptic labeling of C1q protein is observed in association with the plasma membrane (**d1**–**d2**) in perisynaptic locations and likely in association with the spine apparatus (**d4**) in different subcellular microdomains. Presynaptic labeling of C1q protein is observed in association with synaptic vesicles (**d3**, **d4**). **e** C1q labeling is observed in glial leaflets ensheathing the axospinous glutamatergic-like asymmetric synapses. C1q protein is also visualized within the glutamatergic-like PSD per se in the postsynaptic compartment. **f** Postsynaptic labeling of C1q within dendritic spines in extrasynaptic locations in association with the plasma membrane near the PSD. Synapses are between arrows. Color-coded arrowheads (purple) point to C1q immunoreactivity. Profiles are pseudocolored for clarity. Ax, axon; Den, dendrite; Sp, spine; Mit, mitochondria; G, glia. Scale bars, 200 nm

Similar to the monkey results, we observed a significant increase in the C1qA levels in aged rats (Fig. 7A, B). The rise in C1qA levels was only evident in animals over 28 months of age and was still variable within this population. Given the controlled environment of these animals, this variation may reflect the natural biological variability of C1qA levels consistent with the observations in macaques.

#### ImmunoEM localization in aged rat layer II/III mPFC

The anatomical subcellular distribution of C1q protein in aged rats paralleled the distribution in aged rhesus macaques. We found extensive postsynaptic labeling of C1q protein within dendritic shafts in close proximity to MOAS profiles (Fig. 7C). We also observed labeling of C1q protein within pre- and postsynaptic axospinous asymmetric inputs in association with synaptic vesicles and within dendritic spine heads in close proximity to the plasma membrane and spine apparatus (Fig. 7D1–D4 and F). Finally, in addition to the neuronal labeling, we also detected dense C1q labeling within glial leaflets ensheathing glutamate-like axospinous synapses (Fig. 7E).

## Discussion

**Fig. 8:**
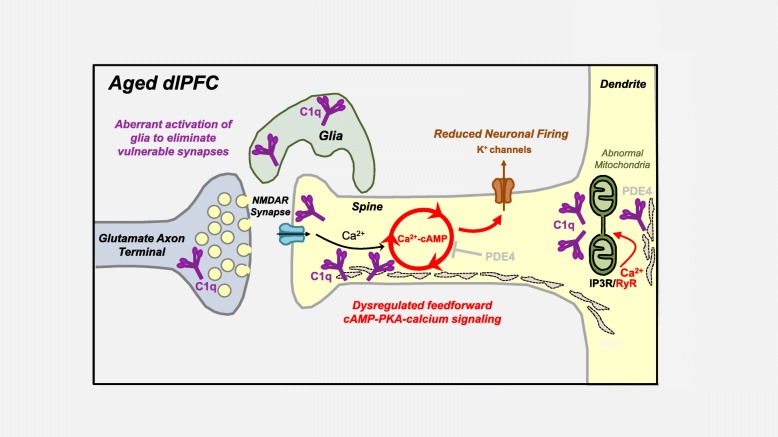
Summary of age-related alterations in complement cascade C1q signaling in dlPFC layer III and hypotheses relating to neuronal pathology. Age-related increases in C1q expression accumulate in the glia and postsynaptically in dendritic spines and dendritic shafts, with a sparser expression in axon terminals. Within dendritic spines, C1q aggregates in perisynaptic and extrasynaptic subcompartments in association with the spine apparatus and within the PSD of glutamatergic synapses. Within dendritic shafts, C1q aggregates in close proximity to dysmorphic mitochondria. We hypothesize that the rise in complement C1q signaling in the aged dlPFC may be due to the age-related dysregulation of feedforward cAMP-PKA-calcium signaling, which increases the open state of nearby K^+^ channels, but may also cause calcium overload of the mitochondria and the initiation of inflammatory actions. The intra-neuronal localization of C1q might signal to microglia and astrocytes, via a mechanism that remains to be delineated, to eliminate dysfunctional neuronal elements and synapses by phagocytosis

The current study characterized the expression and localization of the initiating complement signaling protein, C1q, in the aging macaque dlPFC and rat mPFC, with a focus on the layer III circuits known to exhibit age-related loss of spines. Consistent with previous studies of the aging rodent and human cortex [16, 34], we found a large increase in the expression of C1q with advancing age in the rhesus monkey dlPFC and corroborated this finding in rat mPFC. However, the localization of C1q may be more complex than suggested by some expression studies of aging rodent AD models, which highlighted the microglia as a critical source of C1q [22]. Our data confirm a dense glial localization of C1q in rat mPFC. However, C1q was also localized within pyramidal neurons, particularly in dendrites and spines (summarized in Fig. 8). C1q was located near the synaptic membrane in the spines, but not axon terminals, consistent with IP analyses demonstrating an age-related rise in C1q’s association with PSD95. A recent study of the hippocampus in an AD mouse model with a humanized tau mutation also detected C1q decorating the perisynaptic membrane in association with the PSD [19], suggesting that the postsynaptic compartment may be an initial target in aging and diseases. While the exact nature of C1q’s association with PSD95 remains unclear, it may be through direct binding between the two proteins or through association with other components of the postsynaptic density that are closely associated with PSD95; the accumulation of C1q preferentially at the synaptic membrane of excitatory synapses remains clear. Altogether, these data suggest that C1q may signal from within neurons to initiate phagocytosis by reactive glia or be internalized within neurons in a subcompartment-specific fashion; specifically, intra-neuronal C1q may guide the degenerative process to ailing synapses.

### Relevance of C1q localization in glial cells

Consistent with previous RNA sequencing and immunohistochemistry studies in rodent and human brain [16, 36, 38, 39], we found significant labeling of C1q within the glia, suggesting that the protein may be intrinsically generated in this cell type in the aging macaque dlPFC and aging rat mPFC. Future studies employing multi-label immunoEM will be required to disentangle whether glial C1q protein expression is selective for microglia, or also present in astrocytes. A1 astrocytes promote inflammation and reduce neuronal survival and are abundant in various neurodegenerative diseases including AD, Parkinson’s disease, and Huntington’s disease [40]. Normal aging induces A1-like astrocytic reactivity, suggesting that astrocytic dysfunction with advancing age might contribute to cognitive decline [41]. Recent evidence purports that classically activated microglia secreting C1q, IL-1α, and TNF can induce reactive A1 astrocytes and that these cytokines are necessary and sufficient to induce this phenotype [40]. Aging-induced upregulation of reactive astrocyte genes was significantly diminished in mice lacking C1q, IL-1α, and TNF [41]. Furthermore, exosomes derived from astrocytes in human AD patients show expression of C1q, suggesting that microglia-mediated induction of C1q expression can further propagate C1q synthesis in astrocytes and release into the extracellular space [42]. These results are also congruent with the notion that the upregulation of cytokines in astrocytes and microglia might exacerbate age-related deleterious processes, e.g., generation of free radicals and oxidative stress [43, 44]. Therefore, C1q signaling might be part of a critical molecular mechanism by which microglia can induce A1 astrocyte reactivity to target aberrant neuronal elements and engulf pre- and postsynaptic compartments [12, 14, 45–47].

### C1q is expressed in glutamatergic synapses in aged monkey dlPFC layer III

Although C1q has previously been seen decorating synapses, the prominence of C1q labeling *within* spines was unexpected. C1q was evident on the calcium-storing spine apparatus and near or within asymmetric (presumed glutamatergic) synapses. The labeling of aged, glutamate-like synapses was consistent with the co-IP data showing C1q associating with PSD95, including an increased association with advancing age. These findings are in harmony with recent studies of the aging hippocampus, suggesting that C1q aggregates near the PSDs of Tau-301S mice and AD patients, correlating with phosphorylated tau and microglial engulfment of synapses [19]. In addition, in situ hybridization and immunocytochemistry studies of various complement proteins, including C1q, have detected expression in pyramidal neurons in the temporal cortex and hippocampus of healthy controls and AD patients [48, 49]. Although the role of C1q on the SER/spine apparatus is unknown, previous studies suggest that calcium might be necessary for C1q target recognition and complement activation [50]. As described below, calcium dysregulation with advancing age may be a precipitating factor in driving the inflammatory cascade in dlPFC circuits, and actions on the spine apparatus may be a key site of initial pathology.

C1q label was also observed on synaptic vesicles within the axon terminal, although to a lesser extent than in spines. The presynaptic axon terminal is a major site for C1q actions in the developing nervous system, allowing activity-dependent pruning of inappropriate synapses [15, 51]. For example, in the developing visual system, C1q is highly expressed in retinal ganglion cell (RGC) axons [15, 51]. The induction of C1q within RGCs is regulated by the release of transforming growth factor-β (TGF-β) from astrocytes [52]. Recent studies in murine synaptosomes suggest that C1q is compartmentalized in the presynaptic part of labeled synapses, where it associates locally with apoptosis markers, cleaved caspase-3 and annexin V [53]. The positive correlation between synaptosomal C1q and cleaved caspase-3 suggests that the extent of caspase-3 activation influences the level of synaptic C1q deposition, driving activity-dependent and complement-mediated synapse loss potentially involving neuronal pentraxin 1 (Nptx1) [53]. Our findings suggest such a mechanism is plausible in aged primate dlPFC layer III microcircuits, where presynaptic elements can be tagged for engulfment as well. However, the predominant localization of C1q near spines and dendrites suggests that pathological events may be primarily initiated by postsynaptic elements in the aging dlPFC.

It is noteworthy that the large rise in C1q in rat mPFC was only seen at extreme ages, which were not examined in studies aiming to dissect microglial vs. neuronal contributions [22]. This may explain the lack of excitatory neuronal expression in this previous work [22]. It is possible that C1q expression within excitatory neurons is a feature of aging primate brains and only becomes prominent in rodents at extreme age (current study) or with humanized genetic alterations [19].

### Potential role of abnormal mitochondria in dendritic expression of C1q

It is noteworthy that C1q was observed in dendritic shafts near abnormally shaped mitochondria, as calcium overload of mitochondria can initiate inflammatory signaling [54]. Dendritic mitochondria are critical for the normal physiology of neurons and directly influence the morphogenesis and plasticity of spines and synapses [55]. For example, increasing dendritic mitochondrial content or mitochondrial activity through homeostatically intact fission/fusion enhances synaptic activity [55]. We have previously described the presence of aberrant “MOAS” mitochondria within dendritic shafts in aged monkey dlPFC, a pattern of alterations that were present in the layer III microcircuits required for higher-order cognition [11]. We have hypothesized that the MOAS phenotype may arise from calcium overload of the mitochondria, due to calcium leak from the SER [11]. This has been shown in the cardiac muscle, where excessive PKA phosphorylation of ryanodine receptors (RyR2) causes calcium leak, leading to calcium overload of the mitochondria and inflammatory response [56]. Thus, the novel localization of C1q in close proximity to MOAS profiles in dendrites might shed light on the initiation of inflammatory cascades in aging neurons [57].

### Signaling pathways that exacerbate C1q synthesis: potential role of feedforward cAMP-PKA-calcium signaling

Why are layer III dlPFC pyramidal cells so vulnerable in aging and schizophrenia? We have hypothesized that higher dlPFC circuits are at increased risk of atrophy because they contain the molecular machinery to magnify feedforward cAMP-PKA-calcium signaling and that dysregulation of cAMP-PKA-calcium signaling with advancing age leads to mitochondrial dysfunction and complement activation, inducing phagocytic elimination of spines and cognitive impairment (Fig. 8). We have shown that cAMP-PKA signaling is disinhibited in the aged dlPFC due to the inhibition/mislocalization of PDE4 proteins [1, 9]. Excessive cAMP-PKA signaling has many detrimental actions, including the aberrant opening of K^+^ channels to reduce neuronal firing [8], phosphorylation of tau [9], and dysregulation of calcium signaling through pRyR2 [58]. In the hippocampal neurons, calcium overload of the mitochondria has been shown to activate inflammatory caspase-3 actions [59], which may be associated with increased levels of C1q [53, 60]. In *Drosophila* neurons, calcium transients were shown to precede C1q upregulation and complement-mediated elimination of dendrites [61]. Similar actions in dysfunctional mitochondria in dlPFC dendrites may initiate C1q expression. Alternatively, C1q may be exclusively synthesized in the microglia, but be taken up by, and localized within, neurons at vulnerable sites. This is an area for future research.

### Implications for neurological and psychiatric disease

Various lines of investigation provide compelling evidence that neuroinflammation, including hyperactive microglial and complement cascade activation, is a cardinal feature of various neurological and psychiatric disorders [13, 17, 62–64]. Initial hypotheses speculated that upregulation of neuroinflammatory pathways might be a downstream consequence, but more recent data suggest that the classical complement cascade and microglia are activated early in the disease process to eliminate excessive synapses [12]. For example, the strongest genetic risk association for schizophrenia is found in the loci near the complement C4 gene, and the levels of C4A expression predict the risk of the disease [21]. Similarly, various components of the classical complement cascade are elevated in neurodegenerative diseases [65]. For example, C1q mRNA is increased by as much as 80-fold in regions affected in AD in postmortem brains, contributing to neuropathological hallmarks including amyloid plaques and neurofibrillary tangles [66]. More recently, in a mouse model of frontotemporal dementia caused by progranulin deficiency, there is a striking elevation in C1q expression in the microglia, resulting in concomitant tagging of dysfunctional synapses by C3 and phagocytosis [67]. Importantly, the morphological phenotype of synapse loss, a conserved feature across neurological and psychiatric disease, can be rectified by novel antibody treatments, providing insight into potential treatment strategies [18, 19]. In aggregate, aberrant activation of neuroinflammatory signaling mechanisms during adolescence might be crucial for excessive spine pruning in schizophrenia [21, 68], and re-activation with advanced aging might drive neurodegenerative pathology [16, 18–20], contributing to synaptic and cognitive dysfunction in diseases. The current data add to this emerging field, suggesting that complement activation within pyramidal cells might contribute to the atrophy of higher cognitive circuits and increases naturally with age [16, 69].

## Conclusions

The rise in C1q signaling with advancing age in the vulnerable PFC is associated with prominent localization within neurons as well as its classic glial expression. The localization near the abnormal mitochondria in dendrites as well as in dendritic spines suggests that C1q may help to direct phagocytic activity to ailing synapses in the aging PFC.

## Data Availability

The datasets generated during the current study are available from the corresponding authors on reasonable request.

## Abbreviations

AD: Alzheimer’s disease
Ax: Axon
cAMP: 3′-5′-Cyclic adenosine monophosphate
co-IP: Co-immunoprecipitation
DAB: 3,3-Diaminobenzidine tetrahydrochloride
Den: Dendrite
dlPFC: Dorsolateral PFC
G: Glia
GAPDH: Glyceraldehyde 3-phosphate dehydrogenase
immunoEM: Immunoelectron microscopy
Mit: Mitochondria
MOAS: Mitochondria-on-a-string
mPFC: Medial PFC
PFC: Prefrontal cortex
PSD: Postsynaptic density
SER: Smooth endoplasmic reticulum
Sp: Spine
